# Development and validation of a multivariable prediction model for infection-related complications in patients with common infections in UK primary care and the extent of risk-based prescribing of antibiotics

**DOI:** 10.1186/s12916-020-01581-2

**Published:** 2020-05-21

**Authors:** Chirag Mistry, Victoria Palin, Yan Li, Glen P. Martin, David Jenkins, William Welfare, Darren M. Ashcroft, Tjeerd van Staa

**Affiliations:** 1 Health e-Research Centre, School of Health Sciences, Faculty of Biology, Medicine and Health, The University of Manchester, Oxford Road, Manchester, M13 9PL UK; 2 Greater Manchester Connected Health City, School of Health Sciences, Faculty of Biology, Medicine and Health, The University of Manchester, Oxford Road, Manchester, M13 9PL UK; 3 NIHR Greater Manchester Patient Safety Translational Research Centre, School of Health Sciences, Faculty of Biology, Medicine and Health, The University of Manchester, Oxford Road, Manchester, M13 9PL UK; 4 Public Health England North West, 3 Piccadilly Place, London Road, Manchester, M1 3BN UK; 5 Centre for Pharmacoepidemiology and Drug Safety, Division of Pharmacy and Optometry, School of Health Sciences, Faculty of Biology, Medicine and Health, The University of Manchester, Oxford Road, Manchester, M13 9PL UK; 6 Utrecht Institute for Pharmaceutical Sciences, Utrecht University, Utrecht, The Netherlands

**Keywords:** Antimicrobial resistance, Clinical risk prediction, Common infections, Cox regression, Risk-based prescribing

## Abstract

**Background:**

Antimicrobial resistance is driven by the overuse of antibiotics. This study aimed to develop and validate clinical prediction models for the risk of infection-related hospital admission with upper respiratory infection (URTI), lower respiratory infection (LRTI) and urinary tract infection (UTI). These models were used to investigate whether there is an association between the risk of an infection-related complication and the probability of receiving an antibiotic prescription.

**Methods:**

The study used electronic health record data from general practices contributing to the Clinical Practice Research Datalink (CPRD GOLD) and Welsh Secure Anonymised Information Linkage (SAIL), both linked to hospital records. Patients who visited their general practitioner with an incidental URTI, LRTI or UTI were included and followed for 30 days for hospitalisation due to infection-related complications. Predictors included age, gender, clinical and medication risk factors, ethnicity and socioeconomic status. Cox proportional hazards regression models were used with predicted risks independently validated in SAIL.

**Results:**

The derivation and validation cohorts included 8.1 and 2.7 million patients in CPRD and SAIL, respectively. A total of 7125 (0.09%) hospital admissions occurred in CPRD and 7685 (0.28%) in SAIL. Important predictors included age and measures of comorbidity. Initial attempts at validating in SAIL (i.e. transporting the models with no adjustment) indicated the need to recalibrate the models for age and underlying incidence of infections; internal bootstrap validation of these updated models yielded C-statistics of 0.63 (LRTI), 0.69 (URTI) and 0.73 (UTI) indicating good calibration. For all three infection types, the rate of antibiotic prescribing was not associated with patients’ risk of infection-related hospital admissions.

**Conclusion:**

The risk for infection-related hospital admissions varied substantially between patients, but prescribing of antibiotics in primary care was not associated with risk of hospitalisation due to infection-related complications. Our findings highlight the potential role of clinical prediction models to help inform decisions of prescribing of antibiotics in primary care.

## Background

Antimicrobial resistance (AMR) is one of the biggest global threats facing modern healthcare and medicine [1, 2]. A recent World Health Organization report highlighted the urgency of this problem, identifying that drug-resistant infections cause at least 700,000 deaths globally a year [3]. This number could rise to 10 million per year by 2050 if no action is taken [4–6]. One driving factor behind the emergence and persistence of AMR is the overuse and misuse of antibiotics [7]. It is not purely a contemporary issue, as government committees in the UK discussed strategies to optimise antibiotic usage more than 20 years ago [8, 9]. Despite this, the way physicians make the decision on whether to prescribe has changed little in that time and is still largely reliant on their immediate assessment of a patient’s symptoms.

Primary care was responsible for prescribing over 80% of all antibiotics in the National Health Service (NHS) in 2017 [10]. Earlier research has examined antibiotic prescribing patterns in primary care in the UK and found that it is heterogeneous regionally and nationally [11–13]. A recent study highlighted that substantial variability exists both within and between general practices and that there are multiple drivers behind the decision to prescribe [14]. Together, this suggests that a more evidence-based approach to decision-making for antibiotic prescribing is required to achieve better patient care. Prescribing based on an objective evaluation of a patient’s risk is a relatively new concept but is gaining popularity. For example, prescribing of statins is now guided by the QRISK algorithm [15], used to estimate a patient’s risk of developing cardiovascular disease in the following 10-year period. Applying a similar approach to antibiotic prescribing could facilitate a more targeted use of a medication that is becoming increasingly ineffective. However, to date, there are no validated risk models for this purpose.

The aim of this study was twofold: first, to develop and validate prognostic models that predict the risk of developing infection-related complications in patients who consult their general practitioner (GP) for a common infection; second, to use these models to investigate whether there is an association between the risk of an infection-related complication and the rate of receiving an antibiotic prescription. Three common infections were investigated: lower respiratory tract infections (LRTI), urinary tract infections (UTI) and upper respiratory tract infections (URTI).

## Methods

This was a retrospective cohort study using data from two sources: the Clinical Practice Research Datalink (CPRD GOLD) and the Secure Anonymised Information Linkage (SAIL) databases, which made up the derivation and validation cohorts, respectively. CPRD GOLD contains longitudinal, anonymised, patient-level electronic health records (EHRs) from general practices in the UK with more than 5 million active patient records representing about 8% of the UK population [16]. SAIL contains data from approximately 80% of general practices in Wales and covers around 75% of the 3 million population [17–19].

The EHRs included clinical diagnoses, prescribed medication, vaccination history, diagnostic testing, lifestyle information and clinical referrals, as well as patient’s age, gender, ethnicity, smoking history and body mass index (BMI). Patient-level socioeconomic information was available through linkage of the postcode of a patient’s residence to the Index of Multiple Deprivation (IMD) [20]. Patient-level IMD was aggregated based on quintiles. Antibiotic prescriptions were determined using the British National Formulary.

The derivation dataset from CPRD comprised routinely collected data from 587 general practices in England from 1 January 2000 to 31 December 2015. Patient-level data from the general practices were linked to hospitalisation data (HES for CPRD GOLD, PEDW for SAIL) containing information on the date of hospital admission and the clinical diagnoses established at and during admission (coded using ICD10 codes). Linked data were available for about half of CPRD practices which are all located in England and for all the SAIL practices. Patients were followed for 30 days after their initial GP consultation to determine if they suffered further complications as a result of their infection. The validation cohort (SAIL) covered 338 general practices in Wales from 1 January 2000 to 15 March 2017.

### Study population

The study population included patients consulting their GP for one of three infections (LRTI, UTI and URTI including coughs, colds and sore throats). READ codes (version 2 for CPRD and version 3 for SAIL) were used to extract EHRs for each infection-related consultation. Code lists used in this study are available on the Clinical Codes Repository [21]. Across both datasets, we restricted the study population to incidental consultations (i.e. no record of previous consultation for these infections or antibiotic prescribing 3 months before). Patients could appear in the dataset on multiple occasions (as separate records) due to the long-term nature of the study. For the development of the clinical prediction models (CPMs), we excluded all patients who were prescribed an antibiotic on the day of their consultation.

### Outcomes

The primary outcome was the time between a patient’s GP consultation and hospital admission due to infection-related complications, with censoring at 30 days. Hospital admissions due to infection-related complications were identified by the ICD10 codes for the primary admission diagnosis, where we considered a broad set of infections (such as hospital admission for LRTI, pneumonia, sepsis); the full list is also available at clinicalcodes.org [21]. Hospital admission (for any reason) was also used as an additional outcome for the study.

### Predictor variables

The full list of potential predictors was derived based on a literature review and discussions with clinical experts; this list is outlined in Table 1.

**Table 1:** List of potential predictors considered for the risk prediction models

Factor	Additional information
Age	Age recorded at the time of consultation categorised into 11 groups: < 5 years, 5–10, 10–15, 15–20, 20–30, 30–40, 40–50, 50–60, 60–70, 70–80, 80+. The exception was for the UTI model where there were no events in the 10–15 category (merged to create a 10–20 years category).
Gender	Male/female
Charlson Comorbidity Index [22]	A score summarising the number and severity of comorbidities affecting the patient. The overall score ranges from 0 to 31 [23] but was categorised into five groups: • Very low—score = 0 or 1 • Low—score = 2 or 3 • Medium—score = 4 or 5 • High—score = 6 or 7 • Very High—score > 7
Socioeconomic status	Determined by linking the postcode of a patient’s residence to the Index of Multiple Deprivation 2010 classification [24]. IMD was categorised into quintiles: IMD 1 (least deprived) to IMD 5 (most deprived).
Ethnicity	Split into two categories: • White and not recorded/unknown • Combined ethnic minorities
Prescriptions (non-antibiotics) in the previous year	The number of non-antibiotic prescriptions the patient received in the previous year. This was categorised into tertiles (low, medium and high) and was done independently for each infection. Antibiotic users were included in this categorisation to allow the model to be extensible to that group, although they were not included in the datasets to which the models were fitted.
Flu vaccinations	A binary value to indicate whether the patient had a flu vaccination in the previous year.
Hospitalisation in the previous year	A binary value to indicate whether the patient was hospitalised in the previous year.
Outpatient referral in the previous year	A binary value to indicate whether the patient had a hospital outpatient referral in the previous year.
Year of consultation	Year in which the initial GP consultation took place.
Season of consultation	• Spring (March to May) • Summer (June to August) • Autumn (September to November) • Winter (December to February)

For all infections, patient data for smoking status and BMI were missing for over 50% of patients; hence, they were not considered during the modelling stage. Imputation would not have been feasible without introducing unnecessary bias [25]. Patients for whom IMD information was not available (0.12%) were also removed from the derivation dataset; this step was not required for the validation dataset as the IMD linkage was complete. For patient ethnicity, white and unknown were combined (following the approach taken by Hippisley-Cox et al. [26]), with the remaining ethnicities forming a category labelled combined minorities.

### Statistical methods

Cox proportional hazards regression models were fitted to the derivation cohort. Patients entered the study following a consultation with a GP for one of the three common infections and were monitored for the following 30 days. Age was categorised into 11 age groups; initial investigation using cubic splines was considered but found to be problematic due to the sharp increases in incidence rates in patients > 50 years, causing the models to overestimate the risk in older patients.

To validate the performance of the models developed in CPRD GOLD, they were applied to the SAIL dataset (geographical external validation). Predictive performance was assessed in terms of discrimination (ability of the models to differentiate those who experienced the outcome from those that did not), using established metrics (*R*^2^ statistic for survival data and the concordance value/C-statistic). Additionally, model calibration was quantified by comparing the observed and predicted risks for decile groups based on the predicted risk of the patient.

Model updating methods were applied (see Supplement 1) to CPMs that were found to be miscalibrated in the validation cohort (SAIL). These updated models were internally validated using bootstrap resampling to correct for in-sample optimism (since we did not have a further independent dataset to perform further geographical external validation). After completing model derivation and updating, there were six CPMs: one for each of the three indications, across both CPRD and SAIL. To investigate antibiotic prescribing according to the predicted risk, the models were applied to all patients (non-antibiotic and antibiotic users) in the relevant dataset (e.g. the LRTI CPRD model to all LRTI patients in the CPRD dataset). Patients were categorised into 10 groups based on their risk level, and the prescribing rate for each group was compared.

The extent of risk-based prescribing of antibiotics was evaluated in the CPRD cohort containing both antibiotic and non-antibiotic users with an incidental common infection by first estimating the risks of infection-related hospital admission. This was based on the predictions by the three development prediction models. The probability of patients who received an antibiotic prescription was then estimated for each of the three common infections. The study considered non-linear relationships between whether patients received an antibiotic and the risk of antibiotic prescribing using fractional polynomial models [26]. The final fractional polynomial model was selected by Akaike Information Criterion (AIC) from the combination of two terms for predicted risk of infection-related hospital admissions including x-2, x-1, x-0.5, log(x0), x0.5, x1, x2, x3. The models were adjusted for the calendar year.

The analysis was done using R versions 3.3.3 to 3.5 [27] depending on the analysis environment used for the two datasets. The ‘survival’ package [28] was used to fit the survival models and estimate hazard ratios (HRs) and 95% confidence intervals (CIs). Other packages used included the ‘pec’ package [29] to calculate survival probabilities, the ‘survminer’ package [30] to check the proportional hazards assumption and the ‘rms’ package [31] for the bootstrap validation. The polynomial analysis was done by R package “mfp” [32].

## Results

A total of 10.8 million incidental consultations for URTI, LRTI and UTI were analysed in this study: 8,110,530 from CPRD and 2,727,646 from SAIL. There were 6,311,321 antibiotic users (58.23%) and 4,526,855 non-antibiotic users (41.77%), with 33,067 events recorded in CPRD and SAIL combined. Comparisons between the two datasets indicate that the validation cohort had a much younger demographic (mean age CPRD = 38.98; SAIL = 29.80), although most other covariates had broadly similar values (Table 2). The disparity in mean age can be accounted for by the SAIL dataset having many more patients in the under 6 age group (e.g. in CPRD, the prevalence of URTIs was 18.6%, whereas for SAIL, it was 32.0%), which serves to reduce the average age of the population. The high level of white and unknown ethnicity is also striking. This is primarily due to the high level of unrecorded data for ethnicity, and the values are in line with other similar studies [33].

**Table 2:** Baseline characteristics of the derivation and validation cohorts (i.e. incidental antibiotic users with no antibiotic prescription at the date of consultation and in previous 3 months)

	CPRD LRTI	SAIL LRTI	CPRD URTI	SAIL URTI	CPRD UTI	SAIL UTI
Total, *N*	1,419,725	466,814	5,717,194	1,963,684	973,611	287,897
Males, *N* (%)	628,695 (44.28)	218,594 (46.83)	2,423,833 (42.4)	878,512 (44.74)	132,359 (13.59)	41,349 (14.37)
Females, *N* (%)	791,030 (55.72)	248,220 (53.17)	3,293,361 (57.6)	1,085,172 (55.26)	841,252 (86.41)	246,458 (85.63)
Median age, years	53	42	33	16	52	41
Age category, *N* (%)						
≤ 5 years	155,053 (10.92)	107,097 (22.94)	1,061,821 (18.57)	628,694 (32.02)	26,824 (2.76)	18,273 (6.35)
6–18	91,898 (6.47)	38,852 (8.32)	1,012,710 (17.71)	399,829 (20.36)	69,958 (7.19)	30,133 (10.47)
18–40	242,421 (17.08)	80,786 (17.31)	1,323,811 (23.15)	432,553 (22.03)	247,607 (25.43)	93,602 (32.52)
41–60	377,157 (26.57)	91,511 (19.6)	1,174,640 (20.55)	252,776 (12.87)	240,361 (24.69)	55,948 (19.22)
61–80	414,346 (29.18)	102,210 (21.9)	920,051 (16.09)	188,988 (9.62)	264,576 (27.17)	55,318 (19.22)
Over 80	138,850 (9.78)	46,358 (9.93)	224,161 (3.92)	60,844 (3.1)	124,285 (12.77)	34,533 (12)
Ethnicity, *N* (%)						
White and unknown	1,376,529 (96.96)	459,587 (98.45)	5,420,768 (94.82)	1,917,158 (97.63)	954,772 (97.14)	283,139 (98.38)
Combined minorities	43,196 (3.04)	7227 (1.55)	296,426 (5.18)	46,526 (2.37)	27,839 (2.86)	4668 (1.62)
Charlson Comorbidity Index, *N* (%)						
Very low	747,870 (52.68)	306,723 (65.71)	3,973,455 (69.5)	1,577,432 (80.33)	576,725 (59.24)	198,433 (68.95)
Low	513,754 (36.19)	123,116 (26.37)	1,446,313 (25.3)	323,548 (16.48)	289,691 (29.75)	65,104 (22.62)
Medium	118,117 (8.32)	27,325 (5.85)	227,013 (3.97)	47,483 (2.42)	78,432 (8.06)	17,459 (6.07)
High	28,968 (2.04)	7078 (1.52)	51,882 (0.91)	11,425 (0.58)	20,807 (2.14)	5028 (1.75)
Very high	11,016 (0.78)	2572 (0.55)	18,531 (0.32)	3796 (0.19)	7956 (0.82)	1783 (0.62)
IMD quintile, *N* (%)						
1—most affluent	307,540 (21.66)	112,734 (24.15)	1,321,579 (23.12)	456,651 (23.25)	230,673 (23.69)	58,126 (20.2)
2	312,919 (22.04)	92,361 (19.79)	1,289,612 (22.56)	372,911 (18.99)	234,671 (24.1)	51,635 (17.94)
3	276,524 (19.48)	95,484 (20.45)	1,128,934 (19.75)	378,739 (19.29)	197,637 (20.3)	57,876 (20.11)
4	271,090 (19.09)	77,055 (16.51)	1,078,634 (18.87)	335,535 (17.09)	174,247 (17.9)	53,226 (18.49)
5—most deprived	251,652 (17.73)	89,180 (19.1)	898,435 (15.71)	419,848 (21.38)	136,383 (14.01)	66,944 (23.26)

The incidence rate of events was low among non-antibiotic users, with the mean rate being 1.71 cases per 1000 person-months in the derivation cohort and 7.49 in the validation cohort. For both datasets, the incidence rate increases with age (for adults) and increasing Charlson Comorbidity Index (Table 3). Most of the hospital admissions for infection-related complications were for LRTI (CPRD, 10.28 cases per 1000 person-months; SAIL, 23.73), followed by UTI (2.09; 7.00) then URTI (1.23; 6.42).

**Table 3:** Counts and incidence rates for events of hospitalisation due to infection-related complications for the non-antibiotic users in both the validation and derivation cohorts

	CPRD LRTI, *N* cases (incidence)^#^	SAIL LRTI, *N* cases (incidence)^#^	CPRD URTI, *N* cases (incidence)^#^	SAIL URTI, *N* cases (incidence)^#^	CPRD UTI, *N* cases (incidence)^#^	SAIL UTI, *N* cases (incidence)^#^
Total events, *N*	1646	1777	3702	7117	249	319
Males	780 (10.76)	902 (24.90)	1783 (1.38)	3797(7.55)	92 (3.40)	121 (11.70)
Females	866 (9.89)	875 (22.64)	1919 (1.12)	3320 (5.48)	157 (1.71)	198 (5.62)
Age category						
≤ 5 years	220 (6.99)	793 (32.06)	1131 (1.56)	4957 (11.41)	9 (1.07)	43 (7.66)
6–18	29 (3.65)	27 (5.99)	253 (0.45)	567 (2.55)	6 (0.56)	15 (2.99)
18–40	79 (3.71)	56 (6.51)	502 (0.80)	469 (2.16)	7 (0.25)	13 (1.14)
41–60	170 (5.33)	103 (11.90)	450 (0.81)	279 (2.32)	18 (0.79)	19 (3.17)
61–80	475 (11.38)	292 (19.74)	662 (1.57)	368 (4.33)	75 (2.72)	72 (8.36)
Over 80	673 (26.15)	506 (37.27)	704 (6.52)	477 (15.74)	134 (6.27)	157 (17.47)
Charlson Comorbidity Index						
1—very low	579 (6.74)	1152 (22.36)	2139 (0.98)	6156 (6.67)	48 (0.70)	136 (4.46)
2	605 (11.19)	379 (22.54)	1014 (1.46)	694 (4.37)	104 (2.99)	109 (10.65)
3	307 (21.07)	171 (36.67)	362 (3.61)	180 (8.68)	49 (4.48)	49 (14.47)
4	108 (27.45)	50 (37.56)	130 (5.77)	59 (11.98)	37 (11.11)	17 (16.25)
5—very high	47 (28.58)	25 (46.26)	57 (7.37)	28 (18.03)	11 (7.96)	8 (18.72)
IMD quintile						
1—most affluent	329 (9.73)	436 (25.36)	762 (1.09)	1946 (7.33)	52 (1.98)	70 (7.76)
2	392 (11.33)	358 (24.39)	744 (1.11)	1451 (7.09)	52 (1.90)	51 (6.23)
3	343 (10.73)	344 (22.53)	723 (1.23)	1290 (6.14)	46 (1.93)	65 (7.23)
4	318 (10.68)	305 (23.61)	769 (1.36)	1105 (5.97)	51 (2.32)	71 (8.00)
5—least affluent	264 (8.83)	334 (22.54)	704 (1.46)	1325 (5.44)	48 (2.46)	62 (5.89)
Ethnicity						
White or unknown	1578 (10.13)	1762 (23.80)	3398 (1.20)	6954 (6.44)	244 (2.12)	317 (7.05)
Combined minorities	68 (15.79)	15 (18.09)	304 (1.72)	163 (5.46)	5 (1.29)	2 (3.31)
Hospitalisation (in previous year)	161 (23.33)	143 (35.97)	236 (4.22)	320 (13.43)	38 (6.06)	51 (20.74)
Outpatient referral (in previous year)	1163 (12.08)	837 (27.07)	2434 (1.53)	2832 (7.22)	194 (2.45)	162 (7.69)
Flu vaccination (in previous year)	920 (14.80)	553 (25.61)	1332 (2.21)	1059 (6.60)	150 (3.60)	146 (11.20)

^**#**^Incidence rates of the number of events per 1000 person-months

### CPM derivation

**Fig. 1:**
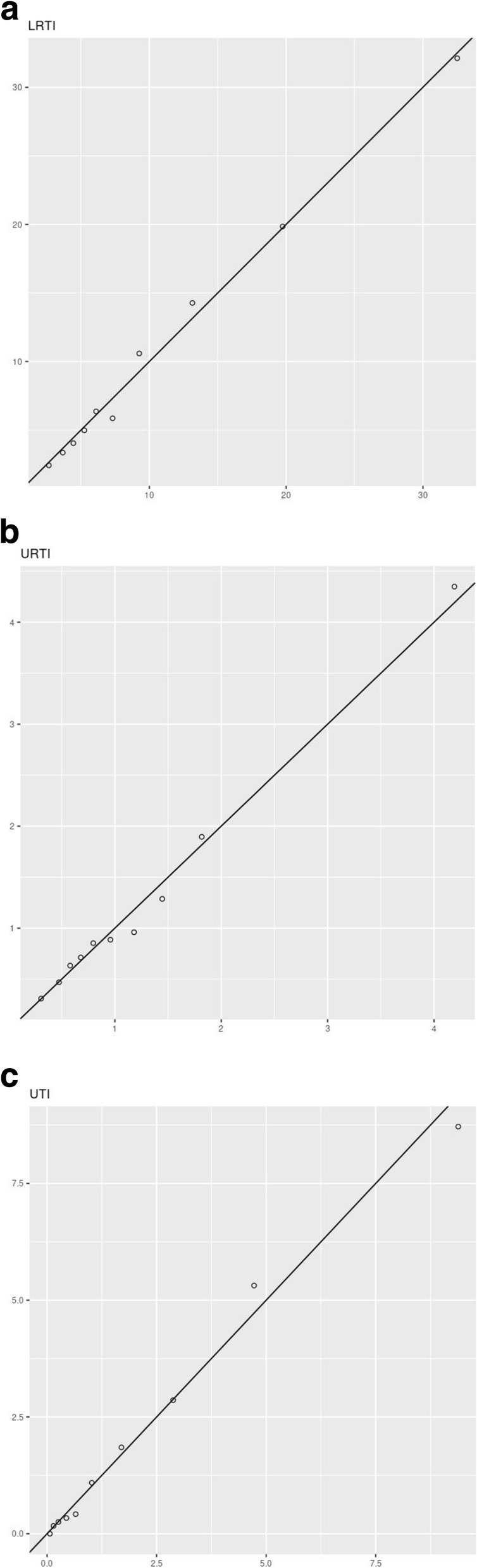
Predicted against observed risks for non-antibiotic users in the derivation cohort (CPRD GOLD) for each decile (stratified by risk level). *x*-axis: predicted risk (*N* events per 1000 person-months). *y*-axis: observed risk (*N* events per 1000 person-months)

After developing the CPMs within CPRD, age proved to be the most influential characteristic in determining the risk level across all infections (Table 4). The HRs were highest for the youngest and oldest patients taking the values of 2.43 (LRTI), 2.20 (URTI) and 10.48 (UTI) for the < 5 category, and 5.76 (LRTI), 4.82 (URTI) and as high as 15.23 (UTI) for the 80+ category. Other factors that had a strong impact on risk were those detailing the patient’s past medical history such as their Charlson Comorbidity Index and previous history of hospitalisation. As expected, within the development cohort (CPRD), the models were well-calibrated (Fig. 1), and the concordance values reported ranged from 0.71 to 0.82 (Table 5).

**Table 4:** HRs for the incidence of hospital admission due to infection-related complications in the derivation cohort (CPRD GOLD)

	LRTI, HR (95% CI)	URTI, HR (95% CI)	UTI, HR (95% CI)
Age category			
< 5	2.43 (1.54–3.82)	2.20 (1.88–2.56)	10.48 (2.20–49.83)
5–10	2.18 (1.22–3.90)	0.67 (0.54–0.83)	9.21 (1.84–46.00)
10–15	1.30 (0.61–2.76)	0.38 (0.29–0.51)	0.88 (0.08–9.76)
15–20	1.21 (0.57–2.56)	0.91 (0.72–1.15)	
30–40	1.35 (0.81–2.27)	1.08 (0.89–1.29)	2.00 (0.37–10.95)
40–50	1.63 (1.00–2.65)	0.93 (0.77–1.12)	3.73 (0.79–17.61)
50–60	1.75 (1.09–2.81)	0.93 (0.77–1.13)	4.05 (0.88–18.65)
60–70	1.85 (1.16–2.95)	1.12 (0.92–1.36)	4.55 (1.02–20.22)
70–80	3.18 (2.01–5.03)	1.70 (1.40–2.06)	9.78 (2.30–41.61)
80+	5.76 (3.67–9.05)	4.82 (4.01–5.78)	15.23 (3.63–63.97)
Charlson Comorbidity Index			
2	1.05 (0.92–1.21)	1.33 (1.21–1.45)	2.08 (1.42–3.05)
3	1.28 (1.08–1.51)	1.76 (1.53–2.01)	1.95 (1.23–3.08)
4	1.43 (1.13–1.80)	2.27 (1.86–2.76)	4.24 (2.57–6.97)
5	1.58 (1.15–2.16)	2.93 (2.22–3.86)	3.10 (1.53–6.27)
Ethnicity			
Combined minorities	2.12 (1.66–2.72)	1.54 (1.36–1.74)	1.03 (0.42–2.51)
Prescription (non-antibiotis) category			
Medium	1.35 (1.13–1.60)	0.86 (0.77–0.94)	2.84 (1.44–5.61)
High	2.09 (1.71–2.57)	1.15 (1.02–1.29)	4.59 (2.22–9.51)
Gender—female	0.81 (0.73–0.89)	0.82 (0.76–0.87)	0.72 (0.56–0.94)
Flu vaccination	0.84 (0.74–0.94)	0.97 (0.88–1.06)	0.69 (0.52–0.92)
IMD quintile			
2	1.10 (0.95–1.27)	0.97 (0.88–1.07)	0.89 (0.6–1.30)
3	1.03 (0.88–1.20)	1.06 (0.96–1.17)	0.88 (0.59–1.31)
4	1.06 (0.91–1.24)	1.19 (1.07–1.31)	1.15 (0.78–1.69)
5	0.87 (0.74–1.03)	1.26 (1.14–1.40)	1.29 (0.87–1.92)
Outpatient referral in previous year	1.07 (0.95–1.20)	1.25 (1.16–1.35)	0.84 (0.61–1.17)
Season			
Spring	1.05 (0.91–1.22)	0.86 (0.79–0.94)	0.81 (0.56–1.17)
Summer	1.10 (0.94–1.30)	0.74 (0.66–0.82)	0.92 (0.64–1.31)
Winter	1.37 (1.20–1.55)	1.03 (0.95–1.11)	1.16 (0.83–1.63)
Year of consultation	1.00 (0.98–1.01)	0.99 (0.98–1.00)	0.99 (0.96–1.02)
Hospitalisation in previous year	1.58 (1.33–1.86)	2.12 (1.85–2.43)	1.59 (1.11–2.26)

**Table 5:** Performance metrics for the prediction models fitted to the derivation cohort (CPRD GOLD)

	LRTI	URTI	UTI
C-statistic (area under curve)	0.719 (se = 0.007)	0.71 (se = 0.005)	0.821 (se = 0.018)
*R*^2^	0.006 (max possible = 0.217)	0.001 (max possible = 0.036)	0.003 (max possible = 0.048)
Likelihood ratio test	1032 on 29 df, *p* = 0	2571 on 29 df, *p* = 0	366.2 on 28 df, *p* = 0
Wald test	1023 on 29 df, *p* = 0	3005 on 29 df, *p* = 0	258.3 on 28 df, *p* = 0
Score (log-rank) test	1218 on 29 df, *p* = 0	3926 on 29 df, *p* = 0	436.9 on 28 df, *p* = 0
Internal bootstrap concordance (C-statistic)	0.719	0.710	0.821

### Model external validation

**Fig. 2:**
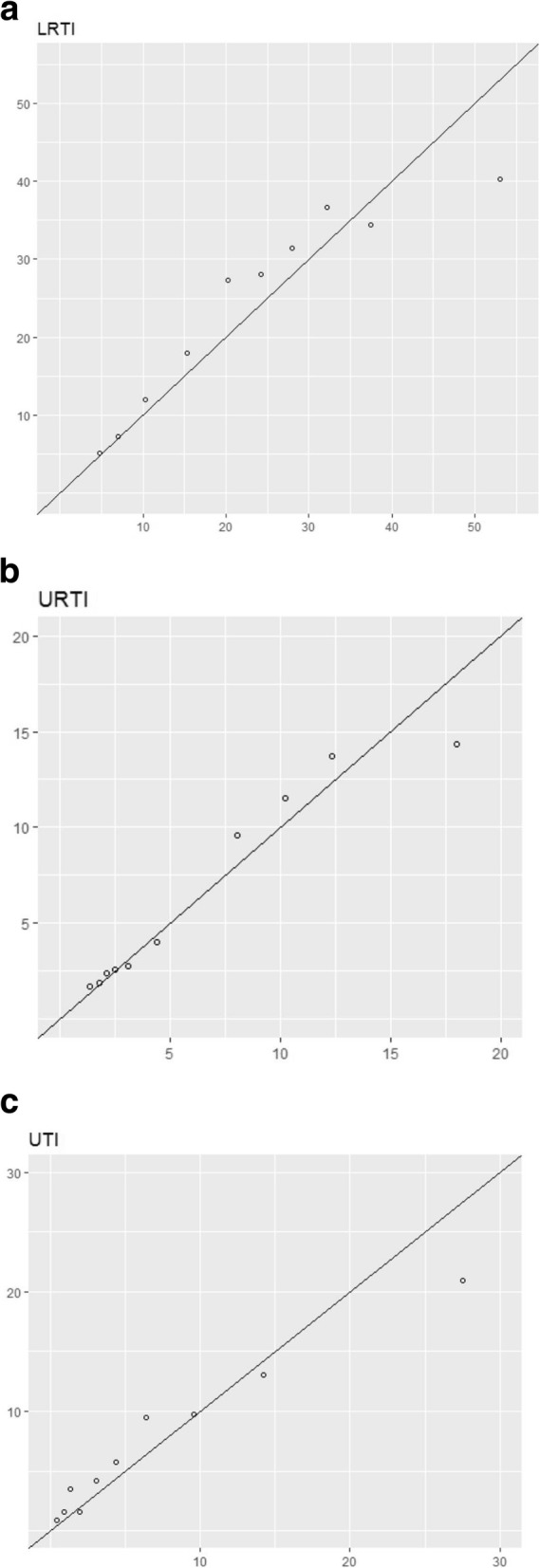
Predicted against observed risks for non-antibiotic users in the validation cohort (SAIL) for each decile (stratified by risk level); models were adjusted for the validation cohort by adding an extra predictor to model age in the derivation cohort. *x*-axis: predicted risk (*N* events per 1000 person-months). *y*-axis: observed risk (*N* events per 1000 person-months)

During first attempts at validation in the SAIL cohort (i.e. geographical validation), the concordance values were 0.61 (LRTI), 0.68 (URTI) and 0.73 (UTI), but poor calibration was observed; the Brier score (averaged over 10 risk groups) was 13.17 cases per 1000 person-months for LRTI (URTI, 5.25; UTI, 4.87). The parameter causing the most divergence when transporting the models was age. Therefore, we updated all of the models to adjust for these differences using an additional age factor (further justification is provided in Supplement 1). Once these models had been refitted (overall adjustment of age shown in Table 6), the calibration was much better (Fig. 2)—Brier score, 3.78 cases per 1000 person-months for LRTI (URTI, 0.92; UTI, 1.76). Bootstrap validation of these models in the validation cohort leads to optimism-corrected concordance values of LRTI, 0.63; URTI, 0.69; and UTI, 0.73. Supplement 2 provides the TRIPOD Checklist for prediction model development.

**Table 6:** Adjusted age HRs following model adjustment in the validation cohort (SAIL)

	LRTI, HR (95% CI)	URTI, HR (95% CI)	UTI, HR (95% CI)
Age category			
< 5	5.47 (2.25–13.26)	5.85 (4.36–7.78)	11.63 (0.97–140.52)
5–10	1.29 (0.40–4.13)	1.71 (1.18–2.47)	3.96 (0.26–58.42)
10–15	1.29 (0.29–5.66)	0.82 (0.50–1.36)	3.27 (0.10–107.46)
15–20	0.76 (0.15–4.02)	1.03 (0.66–1.62)	
30–40	1.24 (0.43–3.61)	1.00 (0.69–1.46)	1.76 (0.10–30.33)
40–50	1.16 (0.41–3.29)	0.97 (0.65–1.42)	1.42 (0.09–22.89)
50–60	1.91 (0.72–5.00)	0.94 (0.62–1.41)	3.00 (0.23–38.79)
60–70	1.81 (0.72–4.66)	1.03 (0.69–1.55)	3.32 (0.28–39.43)
70–80	2.45 (0.98–6.09)	1.65 (1.12–2.41)	4.40 (0.41–47.44)
80+	4.03 (1.65–9.77)	4.29 (3.05–6.01)	7.62 (0.76–78.04)

### Antibiotic prescribing rates

**Fig. 3:**
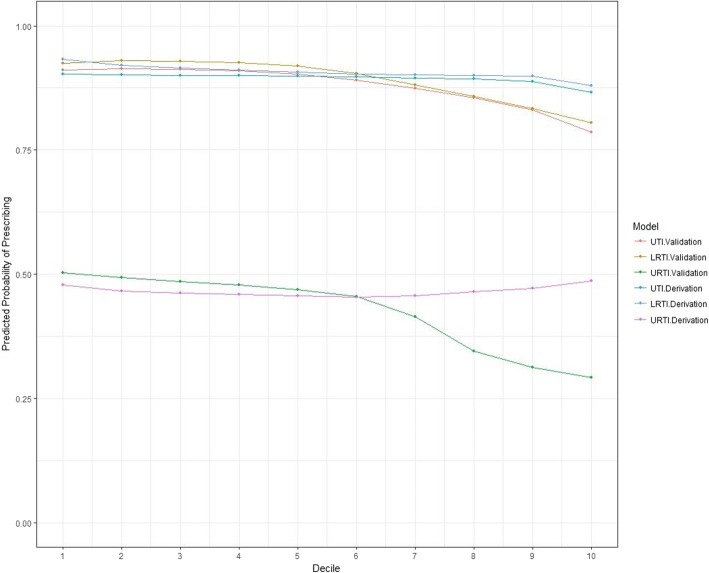
Probability of antibiotic prescribing stratified by predicted risk level for both the derivation and validation cohorts (o = LRTI – derivation cohort; Δ = LRTI – validation cohort; ◼ = URTI – derivation cohort; + = URTI – validation cohort; □ = UTI – derivation cohort; * = UTI – validation cohort). *x*-axis: decile of predicted risk. *y*-axis: probability of antibiotic prescribing

The probability of antibiotic prescribing was plotted for each of the 10 stratified groups of predicted risk for each infection and dataset (Fig. 3). Prescribing rates remained relatively constant across all levels of predicted risk. For all three infection types, patients with very low risks of being hospitalised for infection-related complications were as likely to be prescribed an antibiotic as those with much higher risks.

Additional sensitivity analysis was done using the outcome of hospitalisation for any reason—application of these models to all patients reiterated this finding showing that prescribing rates were relatively uniform across all risk groups.

## Discussion

This study developed three clinical prediction models to predict the risk of infection-related hospitalisation following a GP consultation for URTI, LRTI and UTI using the population-based CPRD and SAIL datasets. The models were successfully adjusted and updated to generalise over datasets covering England and Wales. The models were then applied to datasets containing both antibiotic and non-antibiotic users, and we observed that the decision to prescribe an antibiotic was independent of the risk of hospitalisation due to infection-related complications. Furthermore, it was found that the risk levels of patients vary significantly both across the patient cohort and by indication, which indicates that risk scores provide enough discrimination between patients to offer a viable alternative to traditional approaches to prescribing largely based on symptoms alone. Together, these two observations suggest a potential way to achieve further optimisation of antibiotic usage in primary care.

In previous work, there have been very few attempts to apply clinical risk prediction modelling to the management of infectious conditions. The prediction models that have been developed in this area have focused on specific resistance strains [34, 35] or specific age groups [36] and have had much smaller patient cohorts compared to the size of the populations considered in this study. The major strength of this work is the utilisation of two large independent population-based EHR datasets (both in terms of volume and timespan) for model development and validation. Moreover, individual risk models were developed for each infectious condition, rather than combining multiple conditions in a single model (as others have done [15, 37]).

In 2016, the King’s Fund in the UK examined the pressures of general practice and highlighted the issues faced by practitioners such as increasing workload, greater complexity of work and pressures to meet strict deadlines [38]. In this context, a clinical risk prediction model to objectively assess a patient’s risk of hospitalisation may be welcomed. Estimated risk scores could be presented to the patient, supporting a shared decision-making approach during the consultation. Additional work is needed to validate the clinical risk prediction models before they could be used in clinical practice, but this work represents the first step towards changing the way GPs assess and treat patients for multiple common infections.

Whilst some may argue that the link between antibiotic prescribing and infection-related hospitalisation is not necessarily causal, it is indisputable that antibiotics are the most effective large-scale treatment for common infections. Hence, ensuring the efficient use of antibiotics in primary care is the easiest way to manage the cases of infection-related hospitalisation downstream.

**Fig. 4:**
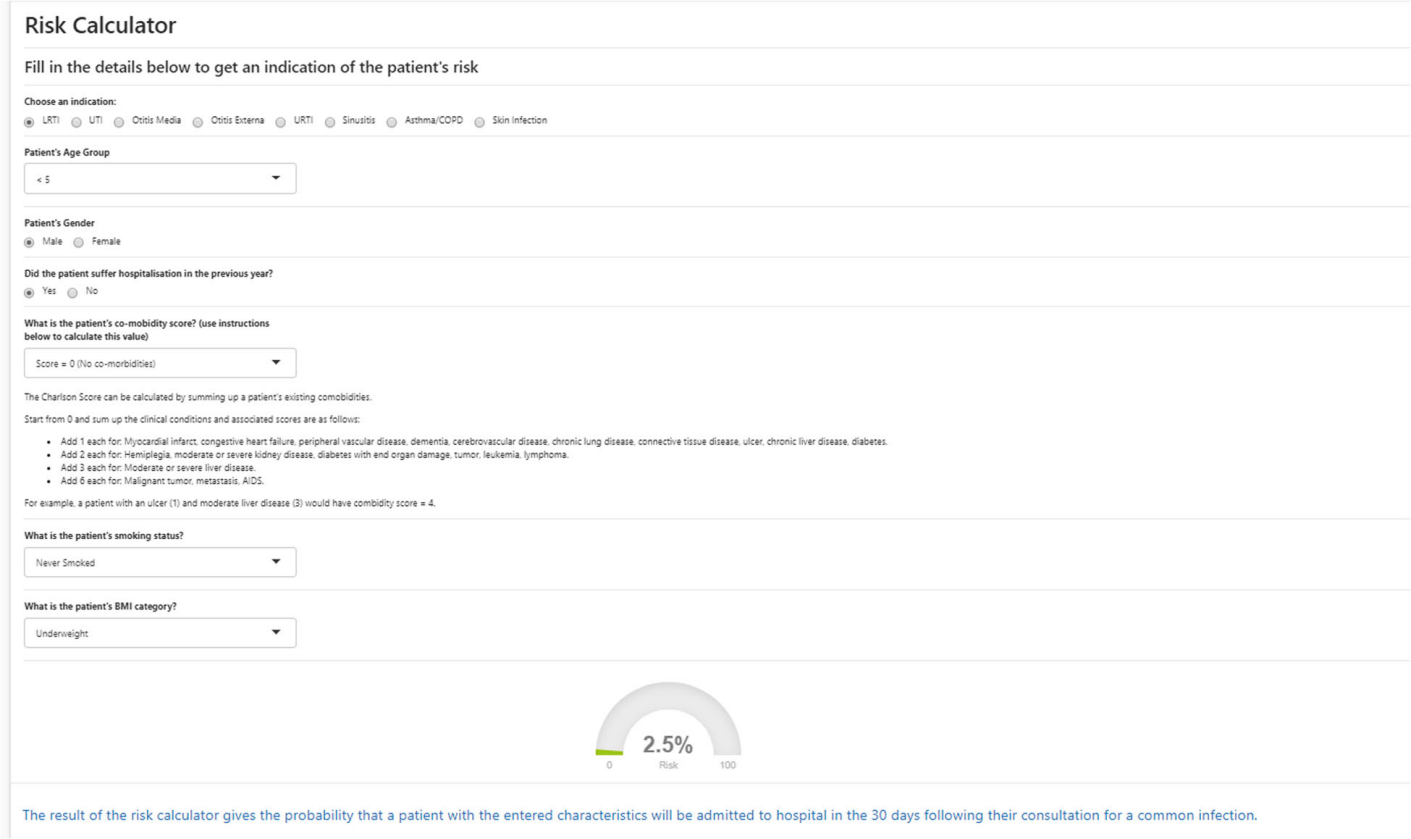
The risk calculator available through the BRIT antibiotic prescribing dashboard

Simplified versions of the models presented here have been made available to medical professionals as an educational resource through a risk calculator tool (Fig. 4) as part of the BRIT Antibiotic Prescribing Dashboard, which is on the HSCN (Health and Social Network). Whilst the models are able to advise on which patients are at the highest risk, they do not explicitly identify which patients should or should not be offered treatment. Given that antibiotics are relatively cheap and very effective, the decision over whether to prescribe is often complicated by the fact that not treating a serious bacterial infection has a much higher cost to the individual than over-treating, leading to physicians prescribing “just in case” [39]. Patients with infections can deteriorate quickly (possibly over a matter of hours), whereas other clinical prediction models investigate conditions (e.g. cardiovascular disease or types of cancer [33, 40, 41]) that develop over a much longer period of time and allow a longer period in which to intervene.

Conversely, it cannot be automatically assumed that antibiotics should only be given to patients with a high risk of infection-related hospitalisation—clinical assessment is a crucial part of the process, and a risk-based judgement is never complete on its own. There are many additional factors that can complicate the decision-making process including whether the infection is genuine, if symptoms will improve with treatment, and potential for infection-related complications. Despite this, the calculators provide a further complementary tool and could work to counteract individual proclivities in prescribing.

Model transportability was a problem during the validation phase of the work, showing that it was difficult to find a model that generalised well to both populations. Domain validation (as done in this study) is notoriously difficult [42] and rarely attempted [43, 44] because it is not easy to account for the demographic contrast and, in this case, local infectious factors. Here, the validation cohort (SAIL) showed a much higher incidence rate of complications overall, in particular, among young children (age < 5), part of which could be attributable to the measles epidemics in South Wales in recent years [45]. Wide CIs were also observed for some groups, e.g. for patients 80+, UTI—this is likely down to the low occurrence of events for this indication, particularly for the baseline group (age 20–30), leading to low statistical power. Despite this difficulty, standard approaches were applied to account for these differences and underpin the results presented, by allowing us to develop CPRD-specific and SAIL-specific models, using the former as the foundations for the latter.

The main limitation with a study of this type is that EHRs can only provide a static snapshot of a patient’s consultation. Because of this, there is no way to fully understand the severity or type of symptoms seen by the practitioner. Here, we separated consultations into three different infections using a single umbrella term to describe many different READ codes, giving the impression that all cases of a single infection have the same level of seriousness, when, in reality, that is not the case. The vast array of codes available and variety by which the same condition can be coded adds to the complexity of conducting this type of analysis [46]. However, despite these limitations, this was the best approach because it would have been very difficult to build the prediction models for individual read codes as the incidence rate would have been too low. Our models were also limited by the selection of the predictors; in particular, some factors were not included. For instance, despite a clear regional variation in primary care antibiotic prescribing [14, 47], this aspect was not built into the prediction models as an explanatory variable. This was done because the intention was to make the model applicable to different regions and ultimately to be used in a clinical setting across the entire UK. Smoking status and BMI, which may be important predictive variables, were also omitted from the models due to large amounts of missing information in the EHRs. Finally, the data used in the study failed to capture instances of delayed prescription or cases where patients do not take (or complete) their prescribed course. These are both interesting subgroups of the main population and would possibly warrant further investigation in their own study.

A recent study investigating the drivers of antibiotic prescribing found that prescribing guidelines alone do not positively influence a change in prescribing, and suggests that more targeted interventions are needed [14]. To achieve the ambitious government target of reducing antibiotic use in the community by 15% by 2024 [48], innovative approaches are required in primary care. Whilst this study represents a start towards that goal, further work is needed including further validation of the models in new datasets and creating new models for other common infections. As well as the research step, it is crucial that this intelligence is available to practitioners to inform their decision-making. An antibiotic prescribing dashboard containing this information is being developed, with the hope of working with general practices to construct a dynamic way to integrate this into point-of-care decision support [49].

## Conclusions

Three clinical risk prediction models were presented, capable of evaluating a patient’s risk of developing further complications as a result of their common infection. The models have been fitted and validated using two large national datasets. Examining prescribing by risk stratification highlighted the lack of relationship between a patient’s risk level and their chance of being prescribed an antibiotic; this is likely due to practitioners prescribing to symptoms but it does show a significant area where improvements could be made to tackle overprescribing of antibiotics.

## Supplementary information

**Additional file 1.** Supplement 1: Details on model validation.

**Additional file 2.** Supplement 2: TRIPOD Checklist for prediction model development.

## Data Availability

The anonymized patient-level data used for this project cannot be shared for reasons of information governance. However, data can be obtained by application to CPRD and SAIL.

## Abbreviations

AMR: Antimicrobial resistance
BMI: Body mass index
CI: Confidence interval
CPM: Clinical prediction model
CPRD: Clinical Practice Research Datalink (data provider for English practices)
GP: General practitioner
HES: Hospital Episode Statistics (hospitalisation data for CPRD)
HR: Hazard ratio
ICD10: International Statistical Classification of Diseases (v10)
IMD: Index of Multiple Deprivation
LRTI: Lower respiratory tract infection
NHS: National Health Service
PEDW: Patient Episode Dataset for Wales (hospitalisation data for SAIL)
SAIL: Secure Anonymised Information Linkage (data provider for Welsh practices)
URTI: Upper respiratory tract infection
UTI: Urinary tract infection
