# Supplementary material for: Development and validation of a multivariable prediction model for infection-related complications in patients with common infections in UK primary care and the extent of risk-based prescribing of antibiotics
Source: BMC Med. 2020 May 21;18:118. doi: 10.1186/s12916-020-01581-2 (PMC7240993; doi:10.1186/s12916-020-01581-2)
Supplement: Supplementary file 1 — Additional file 1. Supplement 1: Details on model validation. [file 12916_2020_1581_MOESM1_ESM.docx]

## Supplementary Information

During validation of the risk prediction models, it was found that the models did not calibrate well when using the CPRD risk prediction models in SAIL. For this reason, the models were recalibrated to the validation dataset (39, 48, 49) and an additional parameter was added to compensate for the different age structure between the two datasets. This resulted in the following formula for the model:


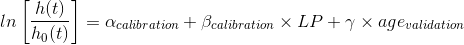


with *LP* representing the linear prediction from the original model, and the final terms on the right-hand side representing the additional age parameter to be fitted. $\beta_{calibration}$ was fixed to equal 1 to avoid minimising the impact of the original model when updating.

It was chosen to add an additional age parameter for the recalibration in the validation dataset after it became apparent that a simple linear adjustment (using an intercept and scaling factor) would not be sufficient to account for the differences in populations. To establish which parameters need to be corrected, separate Cox models were fitted directly to the validation cohort, and the beta coefficients compared by computing the relative difference between each pair of values. For each of the three models, it was found that the coefficients relating to age had the largest discrepancies, corroborating what is seen in Table 3 where the incidence rate of adverse outcomes is very different in young children and the elderly.

### Sensitivity Analysis

To further confirm the findings over the lack of association between patient risk level and prescribing rates, the outcome of hospitalisation for any reason was considered. Following a similar method to the one outlined in the main paper, Cox proportional hazards were fitted to that outcome in the CPRD dataset first, before updating to fit the SAIL datasets. The results of investigating how prescribing changes with stratified risk level are shown in Figure S1.

**Figure S1:** Ratio of prescriptions by stratified risk level for both the derivation and validation datasets using model fitted to calculate the risk of any hospitalisation as the outcome (o = LRTI – Derivation Dataset, Δ = LRTI – Validation Dataset, ◼ = URTI – Derivation Dataset, + = URTI – Validation Dataset, □ = UTI – Derivation Dataset, * = UTI – Validation Dataset). As when investigating the outcome of hospitalisation due to infection-related complications, the results show relatively constant levels of prescribing across all risk levels highlighting a possible way to further optimise antibiotic prescribing.


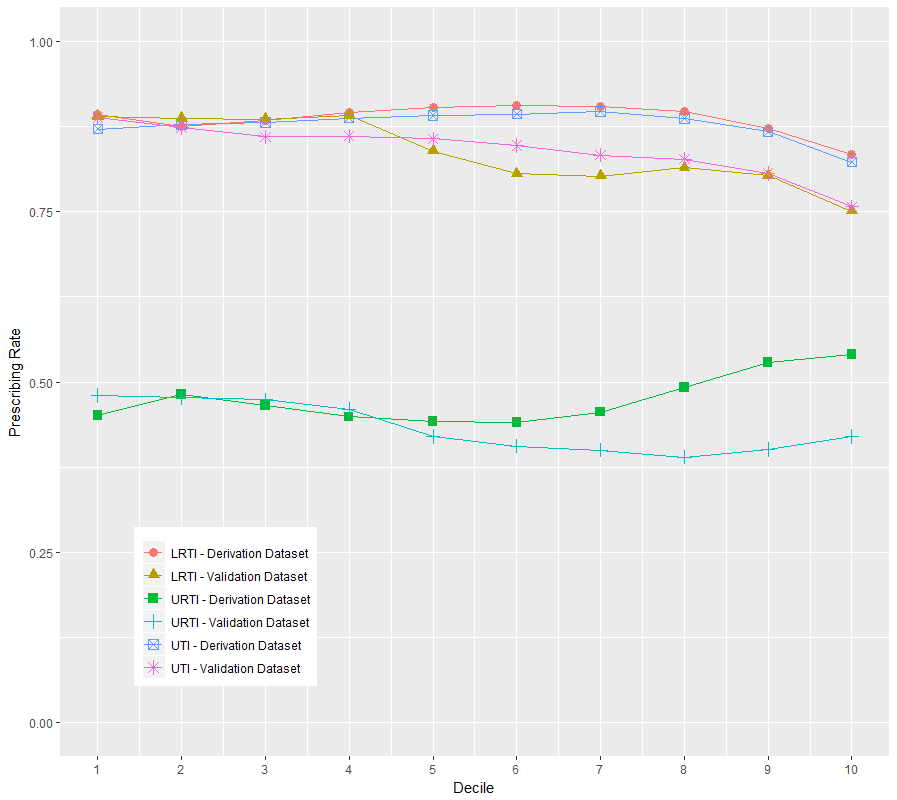


Figure S1: Rate of prescribing by stratified risk level for both the derivation and validation datasets, using prediction models that investigate the outcome of any hospitalisation. Each point on the graph shows the prescribing rate (number of prescriptions / number of patients) for a particular decile (tenth) of predicted risk after applying the risk prediction models to all patients with a particular infection type.
